# Combined chloroquine, sulfadoxine/pyrimethamine and primaquine against *Plasmodium falciparum *in Central Java, Indonesia

**DOI:** 10.1186/1475-2875-5-108

**Published:** 2006-11-14

**Authors:** Edith R Lederman, Jason D Maguire, Iwa W Sumawinata, Krisin Chand, Iqbal Elyazar, Lusi Estiana, Priyanto Sismadi, Michael J Bangs, J Kevin Baird

**Affiliations:** 1 U.S. Naval Medical Research Unit No.2, Jakarta, Indonesia; 2 LITBANGKES (National Institutes of Health Research and Development), Jakarta, Indonesia; 3 District Health Office, Purworejo, Central Java, Indonesia; 4 Poxvirus Program, Centers for Disease Control and Prevention, Atlanta, GA, 30329, USA

## Abstract

**Background:**

Chloroquine (CQ) or sulfadoxine-pyrimethamine (SP) monotherapy for *Plasmodium falciparum *often leads to therapeutic failure in Indonesia. Combining CQ with other drugs, like SP, may provide an affordable, available and effective option where artemisinin-combined therapies (ACT) are not licensed or are unavailable.

**Methods:**

This study compared CQ (n = 29 subjects) versus CQ + SP (with or without primaquine; n = 88) for clinical and parasitological cure of uncomplicated falciparum malaria in the Menoreh Hills region of southern Central Java, Indonesia. Gametocyte clearance rates were measured with (n = 56 subjects) and without (n = 61) a single 45 mg dose of primaquine (PQ).

**Results:**

After 28 days, 58% of subjects receiving CQ had cleared parasitaemia and remained aparasitaemic, compared to 94% receiving CQ combined with SP (p < 0.001). *Msp-2 *genotyping permitted reinfection-adjusted cure rates for CQ and CQ combined with SP, 70% and 99%, respectively (p = 0.0006).

**Conclusion:**

Primaquine exerted no apparent affect on cure of asexual stage parasitaemia, but clearly accelerated clearance of gametocytes. CQ combined with SP was safe and well-tolerated with superior efficacy over CQ for *P. falciparum *parasitaemia in this study.

## Background

Chloroquine (CQ) was the mainstay of antimalarial therapy from 1946 until the past decade when parasite resistance rendered it clinically ineffective in most areas of the world. Adult CQ therapy costs less than $0.20, is widely available almost anywhere in endemic areas, and has a generally good safety and tolerability profile. Resource-strapped health agencies in endemic areas struggle with the decision to abandon this drug. Alternative drugs, such as artemisinin combination therapies (ACT), carry significant limitations linked to cost, ease of compliance and safety in vulnerable populations like infants and pregnant women. Combining antimalarials of differing mechanisms of action may diminish risk of onset of parasite resistance[1], but there are many other determinants of effectiveness and the more effective the therapeutic options available, the more likely treatment will result in a favorable clinical outcome.

CQ combined with sulfadoxine-pyrimethamine (SP) has been proven safe and effective in clinical trials (Table 1) and has been adopted as the primary therapy for uncomplicated falciparum malaria in several countries. Despite the slight increase in cost of using two medications (from approximately $0.10 to $0.20), CQ combined with SP was proven cost-effective [2]. In some nations, this combination is first-line therapy (e.g. Papua New Guinea, Vanuatu, Philippines, Uganda, and Ethiopia). Combining CQ and SP offers compelling advantages in places like Indonesia, principally because both drugs are licensed, widely available, familiar to providers and patients, inexpensive, relatively safe, well tolerated, and easily administered. Furthermore, CQ + SP combined therapy was recently adopted by Papua New Guinea[3] where the level of drug resistance is on par with the most drug resistant areas of Indonesia. Adoption of CQ + SP combined therapy for *Plasmodium falciparum *would require little more than a modification of malaria therapeutic policy and practice. In contrast, most other combined therapy options would require licensing, acquisition, socialization, and carry unfamiliar and perhaps more severe risks linked to safety or compliance. This is an especially salient problem for pregnant women and young children, for whom almost no safety data exists.

**Table 1:** Comparison of clinical trials to evaluate the efficacy of chloroquine (CQ) + sulfadoxine/pyrimethamine (SP)

**Study Location**	**Year**	**N**	**CQ Efficacy**	**SP Efficacy**	**CQ + SP Efficacy**	**Endpoint in Days**	**Comparison of CQ+SP vs. CQ, CQ+SP vs. SP**	**Reference**
Nigeria	2004	153	n/a	n/a	90%	28	n/a	[30]

Laos	2003	110	n/a	n/a	93%	42	n/a n/a	[14]

Bangladesh	2002	133	n/a	n/a	72%	28	n/a	[18]

Eastern Uganda	2002	280	n/a	42%	60%	28	n/a p = ns	[21]

Southern Uganda	2002	448	n/a	70%	83%	14	n/a p < 0.001	[12]

Western Uganda	2001	141	93%	100%	100%	14	p = ns p = ns	[13]

Southern Laos	2001	119	55%	82%	83%	14	p = 0.029 p = ns	[19]

Papua, Indonesia	1999	169	17%	79%	62%	28	p < 0.001 p = 0.047	[17]

Southwest Nigeria	1999	111	93%	n/a	96%	28	n/a p = ns	[22]

Papua New Guinea	1998–1999	513*	n/a	n/a	95%	28	n/a	[3]

The Gambia	1995	405	n/a	90%	95%	28	n/a p = ns	[20]

Mumbai, India	N/a	49	36%	n/a	87%	42	p < 0.026 n/a	[2]

ns = not significant

n/a = not available

* children were treated with amodiaquine + SP (303 of the 513), ** excluding children

Clinical data for CQ combined with SP is still lacking in many countries, including Indonesia, where CQ and SP-resistant *P. falciparum *is prevalent [4-6]. This clinical trial evaluated the efficacy of CQ combined with SP in Central Java, a setting where 47% of *P. falciparum *infections are resistant to CQ and 22% resistant to SP [4]. Since single dose primaquine is mandated on Java and Bali for transmission blocking activity (only adds an additional $0.05 to the cost of therapy), a single dose of primaquine was added to evaluate its impact on parasitological cure and on post-treatment gametocytaemia.

## Materials and methods

### Study location

The study was conducted between July and October 2001 in Purworejo District in the Menoreh Hills near the southern coast of Central Java, a region experiencing resurgent malaria at that time. The social, economic and demographic characteristics of malaria in the region are described elsewhere [4,7,8]. Falciparum and vivax malaria (approximately 1:1) occurs among all ages with prevalence in high-risk areas ranging from 5% to 38%. Among 40 and 54 subjects treated with CQ or SP for uncomplicated falciparum malaria in this region, 47% and 22%, respectively, developed recurrent parasitaemia within 28 days [4]. During the close study follow up, none of the treatment failures developed complicated or severe malaria.

### Study subjects, screening and enrollment

**Figure 1:**
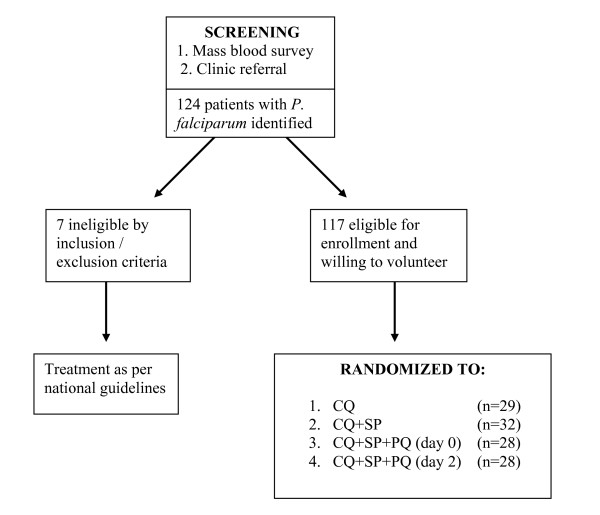
The recruitment, enrollment and randomization process for this study evaluating four treatment regimens for uncomplicated *P. falciparum *in residents of Purworejo District, Central Java, Indonesia.

Subject enrollment is depicted in Figure 1. Subjects with uncomplicated falciparum malaria were recruited, in part, by mass blood survey and by passive case detection (PCD) at local government-run clinics. This was necessary because few adults in this study area suffered from symptomatic malaria. After informed consent, all subjects were screened by medical history, physical examination, semi-quantitative glucose-6-phosphate dehydrogenase (G6PD) assay (G-6-PDH Screening Test 203-A, Sigma Diagnostics^®^, St. Louis, MO USA), and if female of child-bearing potential, a urine human chorionic gonadotropin (hCG) test (TestPack^® ^+Plus™ hCG Urine, Abbott, USA). Inclusion criteria included age ≥ 15, asexual stage *P. falciparum *parasite density ≥ 400/μl on Giemsa thick smear examined by standard microscopy, and availability for 28-day follow-up. Exclusion criteria included pregnancy, breast feeding, body weight < 40 kg, G6PD deficiency, history of antimalarial or antibiotic consumption during the previous 7 days, severe or complicated malaria, history of allergy or adverse reaction to study medications, and *P. vivax *or mixed species infection. Any parasitemic potential study subject not meeting these criteria was referred back to the cooperating clinic for standard therapy. Those patients deemed to meet enrollment criteria and willing to participate were assigned a sequential study subject code by the screening physician. The study subject codes were pre-assigned to treatment arms by a random process and all treatment was pre-packaged accordingly.

### Treatment and follow-up

Subjects were randomized to one of four treatment arms: (A) CQ 25 mg base/kg (body weight) over three days, (B) CQ 25 mg/kg over three days + SP 25/1.25 mg/kg single dose, (C) CQ 25 mg/kg over three days + SP 25/1.25 mg/kg single dose + primaquine 45 mg single dose on day 0, or (D) CQ 25 mg/kg over three days + SP 25/1.25 mg/kg single dose + primaquine 45 mg single dose on day 2. Study investigators directly observed and documented administration of each dose of medication: CQ (Resochin^®^; chloroquine diphosphate tablets 150 mg base; PT Bayer, Indonesia) in three once daily doses of 10, 10 and 5 mg base/kg on days 0,1, and 2 respectively; SP (Fansidar^® ^tablets; 500 mg sulfadoxine/25 mg pyrimethamine; Hoffman La Roche, Indonesia) in a single dose on day 0; Primaquine phosphate (generic tablets, 15 mg base; PT Kimia Farma, Bandung, Indonesia) in a single dose of 3 tablets on day 0 or day 2.

Subjects were followed for 28 days after initiating therapy. Study personnel visited subjects on 10 separate days (1, 2, 3, 4, 7, 11, 14, 18, 21 and 28) to assess symptoms, clinical recovery, adverse events and to obtain finger-stick blood samples for preparation of Giemsa-stained blood smears. Subjects complaining of illness on any day of follow-up were immediately brought to clinic and evaluated. Blood blot specimens (100 – 200 μl) were also prepared on days 0, 2, 7, 14, 21, 28 or day of recurrent parasitaemia from fingerstick blood samples collected by heparinized, 100 μl micro-capillary tubes and expelled onto Whatman No. 1 filter paper (Whatman International, United Kingdom) for drying and storage for later analysis.

### Microscopy

The microscopists in this study were certified as competent by a standardized, proctored examination. Microscopists examined all ocular fields of standard Giemsa (Sigma, USA) stained thick smears at high power (1000× oil immersion) for asexual and sexual forms of *P. falciparum *and other plasmodial species, and recorded sexual and asexual parasite densities as number of parasites per 200 white blood cells. Parasite densities are reported as parasites/μL assuming an average white blood cell count of 8,000/μl.

### Merozoite Surface Protein-2 (*msp-2*) genotyping

*Msp-2 *genotyping was performed on blood blot samples from day 0 and day of recurrent *P. falciparum *parasitaemia following Felger et al. [9]. Recrudescence was defined by the presence of the same genotype on day 0 and day of recurrent parasitaemia while a new infection (i.e. reinfection) was defined by the presence of a different genotype on day 0 and day of recurrent parasitaemia.

### Determination of therapeutic outcomes and data analysis

Therapeutic efficacy (parasitological cure) was defined as the proportion of subjects reaching day-28 without recrudescence. Therapeutic failures occurred when asexual parasitaemia increased between day 0 and day 2, failed to clear by day 4, or recurred between days 4 and 28. These data were used to determine the crude therapeutic efficacy of each regimen. Infections initially classified as therapeutic failures but later proven to be reinfections by *msp-2 *genotyping were reclassified as therapeutic successes with abbreviated follow-up (until the point of censure). These results were used to determine the "adjusted" efficacies of CQ and CQ + SP. EpiInfo 2000 1.1.2 (CDC, Atlanta USA) and SPSS version 9.0 (SPSS, Inc. USA) were used to calculate confidence intervals for odds ratios (OR) and the 95% confidence interval around those ratios, and for estimating the p-value using the Yates' corrected or Fisher's exact test when appropriate. Cumulative incidence of therapeutic failure was estimated by actuarial analysis as described elsewhere [10].

## Results

### Enrollment

Among 124 parasitaemic persons identified during mass blood screening and passive case detection from outpatient clinics, 117 were enrolled and randomized to one of the 4 treatment regimens. Seven subjects did not meet study eligibility criteria and were returned to the referring clinic for treatment for the following reasons: taking quinine (1), pregnancy (1), G6PD deficiency (1), low body weight (2), declined participation (1) and administrative error (1).

### Demographics

Among the 117 subjects enrolled and randomly assigned, no statistically significant demographic differences between the four study arms were identified; age ranged from 16 to 65 years, and the male to female ratio was 1.2:1 (Table 2). No statistically significant differences were noted between treatment arms with regards to fever rates (range 24–41%) or geometric mean asexual parasite densities (range 653-1465/μL) before enrollment.

**Table 2:** Demographic, clinical and parasitological characteristics of subjects treated with 4 malaria regimens in Purworejo District, Indonesia

	**Treatment group**				**Summary statistic**
		
	**CQ alone**	**CQ + SP**	**CQ + SP + PQ0**	**CQ + SP + PQ2**	
**Sample size**	29	32	28	28	n/a
**Male: Female**	1.2 : 1	1.5 : 1	1 : 1.2	1.3 : 1	p = 0.771^a^
**Mean age (range)**	36 (17–55)	40 (20–60)	40 (20–62)	35 (16–65)	p = 0.256^b^
**Geometric mean asexual parasite density (95% CI)**	653 (382–1118)	1465 (849–2531)	1148 (582–2263)	1409 (720–2760)	p = 0.286^b^
**Fever Rate**	24%	41%	29%	36%	p = 0.529^a^

CQ = chloroquine, SP = sulfadoxine/pyrimethamine, PQ0 = primaquine on day 0, PQ2 = primaquine on day 2, 95% CI = 95% confidence interval

n/a – not applicable

a) Chi-square test

b) Kruskal-Wallis test

### Parasite and fever clearance times

**Figure 2:**
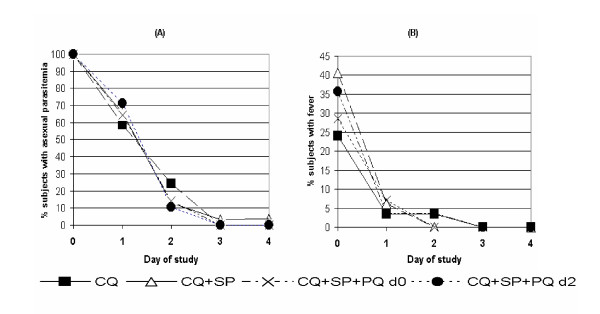
(A) Asexual parasite clearance time in 117 subjects with falciparum malaria after treatment with one of 4 different regimens. (B) Time of fever clearance in 38 subjects with fever at the time of study enrollment. CQ = chloroquine, SP = sulfadoxine/pyrimethamine, and PQ = primaquine on either day 0 (d0) or day 2(d2) of therapy.

There were no significant differences in parasite or fever clearance times among the 4 treatment groups. Virtually all (115 of 117) subjects cleared parasitaemia by day 3 regardless of the therapy assignment, and all 38 subjects with fever prior to therapy had a normal axillary temperature by day 2 (Figure 2).

### Crude therapeutic efficacy of CQ vs. CQ + SP

Asexual parasitemia outcomes were similarly efficacious among the three groups receiving (CQ+SP, CQ+SP+PQ0, and CQ+SP+PQ2), so these groups were combined specifically for comparison with CQ monotherapy against asexual blood stages.

Among the 29 subjects treated with CQ alone, 1 (3%) withdrew from the study after day 18 and 3 (10%) developed intercurrent *P. vivax *during the second and third week of post-therapy observation. Fourteen (48%) completed the study without recurrent parasitaemia. The remaining 11 (38%) subjects developed recurrent *P. falciparum *during the 28-day follow-up. The crude cumulative incidence of failure of CQ therapy for uncomplicated *P. falciparum *was 42% (Table 3); therefore, the crude therapeutic efficacy was 58%.

**Table 3:** Life Table representation of cumulative incidence of crude therapeutic failure attributable to drug resistance among subjects treated with chloroquine (CQ) and CQ + sulfadoxine/pyrimethamine (SP) without distinguishing recrudescence from reinfection

**CQ**						**CQ+SP**					
**D**	**N**	**I**	**W**	**IR**	**CIF**	**D**	**N**	**I**	**W**	**IR**	**CIF**

0	29	0	0	0.00	0.00	0	88	0	0	0.00	0.00
2	29	0	0	0.00	0.00	2	88	0	2	0.00	0.00
4	29	0	0	0.00	0.00	4	86	1	1	0.01	0.01
7	29	0	0	0.00	0.00	7	84	1	0	0.01	0.02
11	29	1	0	0.03	0.03	11	83	0	0	0.00	0.02
14	28	2	1	0.07	0.10	14	83	0	0	0.00	0.02
18	25	2	1	0.08	0.18	18	83	0	2	0.00	0.02
21	22	2	2	0.10	0.26	21	81	0	1	0.00	0.02
28	18	4	0	0.22	0.42	28	80	3	1	0.04	0.06

N = sample at risk of therapeutic failure at start of interval

*i *= the number of cases of therapeutic failure during the interval

*w *= the number of subjects withdrawn from the test during the interval due to exclusion from analysis, loss to follow-up after latest blood smear, intercurrent infection with another plasmodia species, or reinfection by different genotype *P. falciparum *or relapse/reinfection by *P. vivax *based on day of recurrent parasitemia CQ +DCQ concentrations

IR = risk of therapeutic failure in the interval, calculated as *i*/(N-*w*/2)

CIF = cumulative incidence of therapeutic failure, calculated as 1-[(1-IR_n_)(1-CIF_n-1_)], where IR_n _is the IR in the current interval and CIF_n-1 _is the cumulative incidence up to the previous interval

Among the 88 subjects treated with CQ + SP, six (7%) withdrew before day 28 (three declined further participation between days two and 21 and three moved away from the study site between days one and 18). One subject (1%) developed intercurrent *P. vivax *(day 22). Seventy-six (86%) completed the study without recurrent parasitaemia. The remaining 5 (6%) subjects developed recurrent *P. falciparum *during follow-up, therefore providing a crude therapeutic efficacy of 94%. The crude 28-day cumulative incidence of failure of CQ + SP therapy was 6% (Table 3). The relative risk of treatment failure with CQ compared to CQ + SP among those who completed the 28-day observation period was 7.13 (95% CI 2.74 – 18.57, p = 0.00003).

### Adjusted efficacy of CQ vs. CQ + SP

Three of 11 apparent therapeutic failures among subjects receiving CQ alone could not be evaluated because *P. falciparum *DNA could not be amplified for *msp-2 *genotyping, leaving eight evaluable recurrent infections. One case of day 28 recurrent parasitaemia was reclassified as a cure with late reinfection based on differing day 0 and day of recurrence *msp-2 *genotypes. From the remaining seven recurrent infections, the parasite genotypes matched and their classification as recrudescence affirmed. The adjusted cumulative incidence of failure of CQ therapy for uncomplicated *P. falciparum *was therefore 30% (therapeutic efficacy 70%) (Table 4).

**Table 4:** Life Table representation of cumulative incidence of adjusted therapeutic failure attributable to drug resistance among subjects treated with chloroquine (CQ) and CQ + sulfadoxine/pyrimethamine (SP) with *msp-2 *genotyping to distinguish recrudescence from reinfection

**CQ**						**CQ+SP**					
**D**	**N**	**I**	**W**	**IR**	**CIF**	**D**	**N**	**I**	**W**	**IR**	**CIF**

0	26	0	0	0.00	0.00	0	87	0	0	0.00	0.00
2	26	0	0	0.00	0.00	2	87	0	2	0.00	0.00
4	26	0	0	0.00	0.00	4	85	1	1	0.01	0.01
7	26	0	0	0.00	0.00	7	83	0	0	0.00	0.01
11	26	1	0	0.04	0.04	11	83	0	0	0.00	0.01
14	25	1	1	0.04	0.08	14	83	0	0	0.00	0.01
18	23	1	1	0.04	0.12	18	83	0	2	0.00	0.01
21	21	1	2	0.05	0.16	21	81	0	1	0.00	0.01
28	18	3	0	0.17	0.30	28	80	0	2	0.00	0.01

N = sample at risk of therapeutic failure at start of interval

*i *= the number of cases of therapeutic failure during the interval

*w *= the number of subjects withdrawn from the test during the interval due to exclusion from analysis, loss to follow-up after latest blood smear, intercurrent infection with another plasmodia species, or reinfection by different genotype *P. falciparum *or relapse/reinfection by *P. vivax *based on day of recurrent parasitemia CQ +DCQ concentrations

IR = risk of therapeutic failure in the interval, calculated as *i*/(N-*w*/2)

CIF = cumulative incidence of therapeutic failure, calculated as 1-[(1-IR_n_)(1-CIF_n-1_)], where IR_n _is the IR in the current interval and CIF_n-1 _is the cumulative incidence up to the previous interval

In the combined CQ + SP groups, one of five recurrent parasitaemias was excluded from analysis because parasite DNA could not be amplified, leaving four evaluable recurrent infections. Based on discordant day 0 and day of recurrence *msp-2 *genotypes, three apparent therapeutic failures were reclassified as successful cures with reinfection. The 28-day cumulative incidence of failure of CQ+SP therapy for uncomplicated *P. falciparum *was therefore only 1% (therapeutic efficacy = 99%) (Table 4). Subjects treated with CQ alone were 25 times more likely to suffer therapeutic failure compared to CQ combined with SP (95% CI 3.3 – 196.06; p = 0.0006).

### Effect of primaquine on gametocytaemia

**Figure 3:**
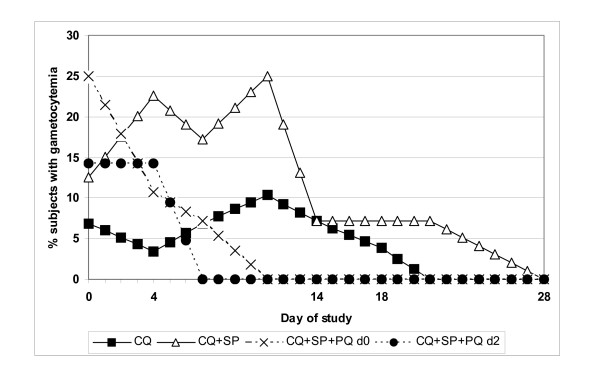
Gametocyte rates in 117 subjects with falciparum malaria after treatment with one of 4 different regimens. CQ = chloroquine, SP = sulfadoxine/pyrimethamine, and PQ = primaquine on either day 0 (d0) or day 2(d2) of therapy.

Figure 3 illustrates the course of gametocyte rates among groups after treatment. Comparing the two groups receiving primaquine, gametocyte rates declined steadily to 0% by day 11 after therapy and did not reappear during follow-up. Gametocyte rates declined more slowly in the non-primaquine groups. The difference in gametocyte clearance rates on day 11 between groups receiving primaquine (0%) and those not (33%) was significant (p = 0.025). A single dose of primaquine greatly improved the clearance rate of gametocytes, and administering the dose on day 2 vice day 0 accelerated clearance time (0% vs. 7% on day 7); however this difference was not statistically significant.

## Conclusion

The combination of CQ+SP proved 99% effective among 87 subjects with uncomplicated falciparum malaria in Central Java, Indonesia. Similarly excellent efficacy has been reported from other regions [2,11-14], including Papua New Guinea[3]. However, in eastern Indonesia, where resistance to CQ (up to 95%)[5] and SP (range 15–54%) [15,16] is much more prevalent than in Central Java, the combination of CQ + SP fails in 38%[17] to 55% (Maguire, personal communication) of subjects with uncomplicated falciparum malaria. Poor efficacy of CQ+SP has been reported elsewhere as well [18] and studies in The Gambia, Uganda, Laos and Nigeria, for example, showed similarly poor efficacy or negligible difference with combining CQ and SP [13,19-22]. The apparent wide range of efficacy of CQ+SP requires information-based decisions about its use as a therapeutic option in any given area.

Despite the marked differences between day-28 cure rates, CQ vs. CQ+SP regimens had essentially identical parasite and fever clearance times. These two indices parallel clinical recovery and almost certainly exacerbate the persistent over-the-counter marketing and unsupervised use of CQ monotherapy. Indeed, surveys of knowledge, attitudes and practices in the region of study[23] affirmed this practice as common. Moreover, CQ remains first-line therapy against vivax malaria, even though accurate diagnostic services to distinguish these species are available to relatively few people. For all of these reasons, CQ remains a widely used drug against *P. falciparum *and other species of malaria in Indonesia.

CQ+SP can not be suggested as a first-line treatment policy that would be supported by comprehensive national programs of patient awareness, health provider training, and widespread distribution and marketing. The efficacy of the combination may not be sufficiently long-lived to support these costly adjustments and measures. However, CQ combined with SP may be a feasible therapeutic option in regions with evidence of treatment efficacy where health care providers are unable to use ACT because of problems with availability, fear of side effects or counterfeit drug. Use should be limited to areas like Central Java, where data suggests that CQ+SP efficacy was better than 95%.

Primaquine has proven gametocytocidal activity against *P. falciparum*. A single 45 mg dose reduces gametocyte clearance time to 2 to 3 days [24], nearly one week faster than with other asexual stage antimalarials alone [25]. The addition of primaquine to standard blood schizonticidal therapy significantly reduced the point prevalence of malaria in one study [26]. Various primaquine regimens appear equally efficacious[27]; however, its use may be limited by the frequency of G-6-PD deficiency and sporadic supplies[28,29]. In our study, addition of a single 45 mg dose of primaquine on either day 0 or day 2 significantly offset the persistence of gametocytaemia. Current *P. falciparum *malaria treatment policy in Indonesia includes administration of 45 mg of primaquine on day 0 of treatment in hypoendemic areas. This study supports its continued use in Indonesia, especially with combination regimens containing SP, which has been linked to sulfonamide-associated gametocyte proliferation [17]. Combining artesunate with SP similarly reduced gametocyte rates below that observed with SP alone in one study in Indonesia[15]; similarly, artesunate combined with amodiaquine significantly reduced gametocyte carriage in comparison to CQ+SP in Nigerian children[30].

In summary CQ+SP combination was highly effective for parasitological cure in subjects with uncomplicated *P. falciparum *in Central Java, Indonesia. The regimen was safe and efficacious, and it is inexpensive and readily available throughout Indonesia. A single dose of 45 mg primaquine, either on day 0 or day 2, suppressed or quickly eliminated gametocytaemia. Given wide regional differences in efficacy of CQ+SP, this regimen should be evaluated where its use is advocated as a therapeutic option for falciparum malaria.

## Authors' contributions

EL and JM are infectious disease specialists and contributed to the data analysis and drafting of the manuscript, and supervision of the field site. JKB is a parasitologist and was the principal investigator of the protocol and senior editor of the manuscript. MJB is an entomologist and contributed to site supervision and manuscript editing. IE is a statistician who performed the data analysis and supervised the data collection in the field. KC, PS, IW and LE are Indonesian general practitioners with experience in caring for subjects with malaria; they performed the screening, recruitment and care of subjects during this study as well as drafting of the manuscript.
